# Computational NEXAFS Characterization of Molecular Model Systems for 2D Boroxine Frameworks

**DOI:** 10.3390/nano12091610

**Published:** 2022-05-09

**Authors:** Daniele Toffoli, Elisa Bernes, Albano Cossaro, Gabriele Balducci, Mauro Stener, Silvia Mauri, Giovanna Fronzoni

**Affiliations:** 1 Department of Chemical and Pharmaceutical Sciences, University of Trieste, Via L. Giorgieri 1, I-34127 Trieste, Italy; toffoli@units.it (D.T.); elisa.bernes@units.it (E.B.); acossaro@units.it (A.C.); balducci@units.it (G.B.); stener@units.it (M.S.); silvia.mauri@phd.units.it (S.M.); 2 CNR-IOM, Istituto Officina dei Materiali, I-34149 Trieste, Italy

**Keywords:** DFT calculations, X-ray absorption spectroscopy, boroxine network

## Abstract

The electronic properties of 2D boroxine networks are computationally investigated by simulating the NEXAFS spectra of a series of molecular models, with or without morphologic defects, with respect to the ideal honeycomb structure. The models represent portions of an irregular 2D boroxine framework obtained experimentally, as supported by the Au(111) surface. The B K-edge NEXAFS spectra are calculated within the transition potential (TP) approximation (DFT-TP). The role of the Au(111) supporting surface on the spectral features has also been investigated by comparing the calculated spectra of a defect-rich model in its free-standing and supported form. The calculated NEXAFS spectra differ from the experimental ones, as the position of the main resonance does not match in the two cases. This finding could suggest the presence of a strong interaction of the 2D boroxine network with the Au substrate, which is not captured in the model calculations. However, good agreement between measured and calculated B K-edge NEXAFS spectra is obtained for a model system, namely, trihydroxy boroxine, in which the B atoms are less screened by the valence electrons compared to the B–B linked boroxine network models considered here. These results suggest catalytic activity in the gold substrate in promoting a weakening or even the breaking of the B–B bond, which is not revealed by calculations.

## 1. Introduction

The 2D single-layered Covalent Organic Frameworks (2D COFs) are a class of systems which have been exploited as interesting templates on metal surfaces to arrange organic guest molecules in regular nano-arrays [1,2,3]. In particular, single layers of boronic-acid-derived COFs have gained attention as novel 2D materials composed of boron and oxygen atoms, which can be grown on metal surfaces and can yield extended porous networks [1,4,5,6,7,8]. The self-condensation of boronic acids on a metallic surface leads to the formation of boroxine rings (B_3_O_3_) and the release of three water molecules, giving rise to boroxine-based COFs which exhibit important properties in terms of crystallinity and thermostability [9]. We recently showed that, starting from phenyl boronic acid as a precursor, the resulting boroxine film on Au(111) can act as an out-of-plane charge transport mediator between the substrate and the organic overlayer [6]. However, boronic COFs exhibit only limited in-plane charge carrier mobility due to a low electronic coupling between boroxine and phenyl rings. An emerging 2D material, related to boronic 2D COFs and possibly overcoming the inner electronic transport issue, is the phenyl-free framework of boroxine rings. Theoretical studies revealed important properties of this system in terms of its transport, optical and mechanical properties [8,10], as well as its intriguing affinity for CO_2_ and drugs, making it a candidate for applications in nanotechnology [11,12,13]. Experimentally, the material was recently synthesized on the Au(111) surface using the tetrahydroxydiboron (THDB) molecule as a precursor [7]. Resulting from the STM (Scanning Tunneling Microscopy) images, the morphology of the network is far from being the ideal long-range ordered hexagonal one. It is rather characterized by a proliferation of defects, with different resulting arrangements among boroxine rings, such as 4-fold, 5-fold, and 7-fold motifs, preventing the formation of a perfect 2D network with a 6-fold motif as the ideal building block (see right side of Supplementary Figure S1). The 2D boroxine framework was also investigated by using Near-Edge X-ray Absorption Fine Structure (NEXAFS), at both the B and O K-edges and, despite the very complex morphology of the boroxine framework and its defective nature, the polarized NEXAFS spectra appear quite simple, being dominated by a strong peak in the *p*-polarization. Considering, in particular, the B K-edge spectrum, significant spectral differences emerged with respect to the NEXAFS spectra of the phenyl-containing boroxine monolayer [6], which clearly indicate the different chemistry of boron in the two systems. More importantly, the B1s spectrum of the boroxine framework differs from that of the bis-catecholato diboron molecule (B_2_Cat_2_) on Au(111) [14], where two resonances are visible in the *p*-polarization. These features were identified as π(B–B) bonding and antibonding transitions through DFT calculations, and considered as a probe of the B–B bond formation on the surface. The presence of a single peak in the boroxinated system of ref. [7] could be, therefore, ascribed to a strong interaction of the monolayer with the substrate, which could involve the B–B bonds connecting the boroxine rings and change the boron chemical environment. To examine the aspects tied to the role of the B–B bond in the THDB-boroxinated system and its relationship with the experimental NEXAFS spectra, here, we present a computational study on the B1s NEXAFS spectra of a series of boroxine-based molecular models, which mimic the motifs suggested in ref. [7] to describe a defective network of B–B-bonded boroxine groups. We focus our attention to the photon energy region corresponding to the main transition as it demonstrates, as it will be shown, the most relevant in terms of the matching of calculated and experimental spectral features.

**Figure 1:**
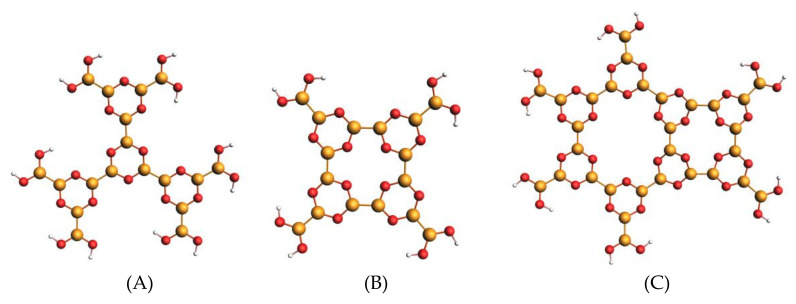
Ball-and-stick representation of the building blocks models of the THDB network: (**A**) model M1, portion of an ideal honeycomb boroxine network, (**B**) model M2, (**C**) model M3. B atoms in yellow, O atoms in red, H atoms in white.

The chosen models for the B–B-bonded boroxine groups are sketched in Figure 1.

Model M1 constitutes the building block of the ideal 6-fold motif of a periodic 2D network (see right side of Supplementary Figure S1); model M2 represents a 4-fold motif, which breaks the order of a regular structure and shapes a possible defect in the network (as suggested by the STM image in ref. [7]), while model M3 contains both the 6-fold and 4-fold motifs and can, therefore, describe the influence of the defects on the NEXAFS spectra. The adsorption of the M3 on the Au(111) surface will also be considered, in order to investigate the role of the molecule–substrate interaction and its effect on the calculated B1s NEXAFS spectra. The THDB molecule, the diboron precursor in the synthesis of the 2D framework, completes the series of molecules with B–B bonds. The computational analysis is further extended to the trihydroxy boroxine molecule (THBoroxine), a system with the B atoms of the boroxine ring not involved in external B–B bonds but in external B–O bonds with the –(OH) terminations.

Density Functional Theory (DFT) with the Transition Potential (TP) approximation (DFT-TP) is employed to calculate the B1s NEXAFS spectra. At the DFT-TP level, the main relaxation effects following the core-hole formation [15,16] are included and good results are generally expected for K-shell excitations of light atoms in the periodic table. The theoretical description of B1s’ core excitations in boronic systems is, however, challenging, as the static correlation effects have to be included in the computational approach, as demonstrated in our previous works [14,17]. Static and dynamic correlation effects can be described by using wave-function-based approaches [17], which are, however, limited to rather small systems. The computationally cheaper ΔSCF-DFT (ΔSCF) method has been demonstrated to be effective for describing the B1s’ core excited states of the diboron system B_2_Cat_2_ [14]. At the ΔSCF level, the relaxation changes among different excited-state configurations relative to the same core-hole are taken into account at variance with the DFT-TP approach, which includes the relaxation in an average way. In this work, we employed the ΔSCF method to calculate the B1s NEXAFS spectrum of the THDB molecule, which is the precursor of the systems here studied and representative of boronic- and B–B-containing molecules. In this way, possible differences with respect to the DFT-TP approach can be analyzed and taken into account in evaluating the calculated NEXAFS spectra of the more-extended models, for which the ΔSCF method becomes impracticable due to convergence problems.

## 2. Methods

### 2.1. Geometry Optimization

Geometry relaxations of M1, M2 and M3 molecular models as well as of M3 adsorbed on the Au(111) surface were performed with the Quantum-Espresso suite of codes [18,19] in the frame of Density Functional Theory with the Kohn–Sham (KS) orbitals expanded in plane waves and the effects of atomic core regions accounted for by pseudopotentials. Ultrasoft pseudopotentials [20] were used throughout the calculations. The exchange–correlation part of the energy functional was modeled with the (spin–unpolarized) generalized gradient approximation (GGA), in the PBE parameterization [21]. Van der Waals interactions were accounted for by use of the vdW-DF functional, available in the Quantum-Espresso suite. The plane wave expansion of the crystalline orbitals was truncated at a cutoff energy of 25 Ry (as determined by convergence of the total energy upon stepwise increase in the cutoff) and a corresponding cutoff of 250 Ry was used for the expansion of the augmentation charge needed by the ultrasoft pseudopotential method. Due to the large unit cells used for the calculations, the first Brillouin zone was sampled at the gamma point only.

For the three isolated molecules, a “molecule box” methodology was applied, where a single molecule is (periodically) simulated in a unit cell which is large enough so as to minimize any interactions between the molecule and its periodic images. Tetragonal unit cells were set up with dimensions large enough to give a minimum separation greater than 10 Å between nearest atoms of any two contiguous periodic images. The supercells containing the investigated molecular models are shown in Supplementary Figure S2. In all the molecular models the external boroxine rings are terminated with the boronic –B(OH)_2_ group.

For the relaxation of the M3@Au(111) system, the “slab method” was used, where an infinite, 2D periodic crystal slab parallel to a given crystallographic family of planes is made periodic along the third space dimension, with the introduction of an additional and sufficiently large vacuum region which ensures that the upper and lower faces of successive slabs do not appreciably interact. An Au crystal slab with the open surface parallel to the (111) plane family was set up by periodic repetition of a supercell consisting of 3 layers of Au atoms. A 10 Å thick vacuum region was added along the z axis (normal to the surface). The so-prepared crystal slab was relaxed before the isolated M3 molecule (previously relaxed) was docked onto it and this starting configuration was finally relaxed to convergence. During the final structural relaxation of the M3@Au model, the coordinates of the two bottom-most Au layers were frozen at their relaxed values before adsorption and those of the upper-most layer were allowed to vary together with the coordinates of the adsorbed M3 molecule. Convergence thresholds for all geometry optimization were 1 × 10^−4^ Ry for total energy and 1 × 10^−3^ Ry/Å for the maximum force component acting on atoms. The unit cell of the M3@Au model is reported in Supplementary Figure S3.

The geometry optimization of THDB and THBoroxine molecules was performed by using the hybrid B3LYP xc potential and the triple ζ polarized (TZP) basis set of Slater-type orbitals (STOs) extracted from the Amsterdam Density Functional (ADF) database [22,23].

### 2.2. NEXAFS Spectra Calculation

For the calculations of the NEXAFS spectra of the M1, M2, M3 and M3@Au systems, suitable finite clusters were cut out from the periodic relaxed structures, previously described, and then used for the calculations of the core excitation energies and oscillator strengths. The side view of the M3@Au cluster is reported in Supplementary Figure S3.

Core excitation energies and oscillator strengths of all finite molecular systems were calculated employing the molecular quantum chemistry ADF program [22,23]. The electronic structure calculations were performed within the scalar relativistic (SR) self-consistent field KS method using the GGA for the xc energy functional with the PW86x Perdew functional [24]. Different basis sets consisting of STO functions were selected to calculate the B K-edge spectra of the systems, taken from the ADF database. For all the systems, an even-tempered quadruple ζ with three polarization and three diffuse functions basis set (designed as ET-QZ3P-3DIFFUSE in the ADF database) was employed to describe the core-excited B atom, while the core orbitals of non-excited B and O atoms were treated by the Frozen Core (FC) technique (TZP.1s basis set); the FC procedure ensures the localization of the core-hole on the excited B atomic site. Furthermore, a TZP basis set was used for the H atoms. For the M3@Au surface cluster, a double ζ (DZ) basis set was employed for H atoms to obtain convergent results. A zeroth-Order Regular Approximation (ZORA) DZ basis set with an FC up to 4f (DZ.4f) was used for all three Au layers; the employment of this basis set was ultimately justified, since the Au atoms interact only weakly with the adsorbed overlayer.

The B1s NEXAFS spectra were calculated with the DFT-TP method [15,16] in which half an electron was removed from the excited B1s core orbital relaxing all the remaining orbitals until self-consistency. The excitation energies correspond to the eigenvalue differences between the final orbitals and the 1s core orbital of the TP configuration:

(1) $$\Delta E_{i\to f}=\epsilon_{f}^{TP}-\epsilon_{i}^{TP}$$

The transition intensities are expressed as oscillator strengths, $f_{i\to f}$. For a randomly oriented (gas phase) molecule, it corresponds to:

(2) $$f_{i\to f}=\frac{2}{3}n_{i}\Delta E_{i\to f}{\left|\langle \varphi_{f}^{TP}|\mu |\varphi_{i}^{TP}\rangle \right|}^{2}$$

while for the adsorbed and fixed-in-space molecule $f_{i\to f}$ reads:

(3) $$f_{i\to f}=2n_{i}\Delta E_{i\to f}{\left|\langle \varphi_{f}^{TP}|\varepsilon \cdot \mu |\varphi_{i}^{TP}\rangle \right|}^{2}$$

where the dipole moment integrals involve the initial and final TP MOs, $n_{i}$ indicates the occupation number of the core orbital in the ground state, and ε is the polarization vector of the incident radiation.

For each non-equivalent B site, a separate calculation of the TP core excitation spectrum was performed and the total B1s spectrum was obtained by summing up the partial contributions weighted for the number of equivalent B atoms. The TP excitation energies are generally overestimated due to the less-attractive potential; therefore, it is usual to shift them with respect to the ΔIP value corresponding to the energy difference $[\epsilon_{1s}^{\mathrm{TP}}-\Delta \mathrm{SCF}(1s)]$, where $-\epsilon_{1s}^{\mathrm{TP}}$ corresponds to the TP Ionization Potential (IP) of the 1s core orbital, while ΔSCF(1s) corresponds to the ΔSCF 1s^−1^ IP energy of the 1s^−1^ ionic state, with the energy of the 1s^−1^ ionic state obtained through an SCF-unrestricted calculation.

The B1s core excitation energies in the THDB and THBoroxine molecules were also calculated at the ΔSCF level with the PW86x Perdew functional [24], in order to better describe the relaxation effects. The ΔSCF-DFT (ΔSCF) core excitation energies were obtained as the energy difference between two self-consistent-field calculations (SCF) relative to the ground state (GS) and the core excited state obtained by the promotion of an electron from the core orbital to one of the unoccupied orbitals in the ground state. The MOs were re-optimized for each excited electron configuration and a different set of MOs for each excited state was calculated within a spin-polarized scheme according to the spin-purification formula [25]. The two sets of Kohn–Sham (KS) MOs, $\left\{\varphi_{\mu }^{i}\right\}$ and $\left\{\varphi_{\mu }^{f}\right\}$, are used to construct the GS initial ($\Psi_{i}$) and final ($\Psi_{f}$) Slater determinants, respectively, which enter in the dipole matrix element for the calculation of the oscillator strengths:

(4) $$\mu_{i\to f}=\langle \Psi_{f}|\hat{\mu }|\Psi_{i}\rangle$$

Since the two sets of MOs are non-orthogonal, the N-electron matrix elements of Equation (4) were evaluated with the general rules for non-orthogonal spin orbitals derived by Löwdin [26]. Further details on the evaluation of the spectral intensities in the ΔSCF approach employed in the present work can be found in refs. [14,27].

Only transitions which occur below the ionization threshold can be accurately described by the employed computational protocol; above it, only qualitative information can be extracted, since the electronic continuum wave function cannot be properly described with standard basis sets of quantum chemistry programs. The ΔSCF B1s IPs were, therefore, reported in all the figures of the NEXAFS spectra.

## 3. Results and Discussion

In the following discussion, the calculated B1s NEXAFS spectra of the THDB precursor and of the M1, M2 and M3, as free molecular models, will be first analyzed in order to assign the spectral features and to discuss their evolution with the increasing molecular complexity of the systems considered. The influence of the Au surface will then be analyzed by comparing the B1s spectrum of the M3@Au cluster with that of the M3 free model. Finally, the comparison between the B K-edge experimental results of the 2D boroxine framework [7] and the calculated B1s spectra of M3@Au cluster and of THBoroxine molecule will be considered to evaluate the reliability of the employed molecular models.

### 3.1. THDB Molecule

**Figure 2:**
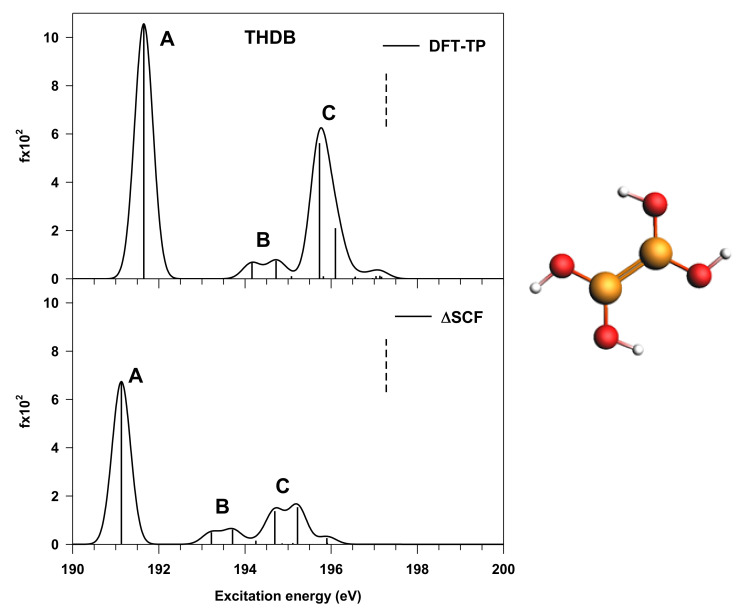
Calculated B K-edge NEXAFS spectrum of THDB (reported on the right side). (**Upper panel**): DFT-TP results; (**lower panel**): ΔSCF results. The stick spectra are broadened by using a Gaussian line shape with FWHM = 0.5 eV. ΔSCF B1s IP (197.28 eV) is reported as a vertical dashed line.

The theoretical results of the B K-edge NEXAFS spectrum of THDB relative to the DFT-TP and ΔSCF calculations are reported in Figure 2.

As one can observe, the spectral features below the edge are preserved in the two approaches, with a main peak (A) around 191 eV, followed by a low-intensity feature (B) generated by two transitions and a more intense peak (C), still contributed by two main transitions. A slight shift towards lower energy in the ΔSCF spectrum compared to the DFT-TP one is found; however, the most significant difference between the two spectra concerns the intensity distribution among the A and C features, with a strong reduction in the intensity ratio between A and C peaks in the case of the ΔSCF results. The incorrect intensity distribution provided by the DFT-TP approach in the case of B1s core excitations already emerged in previous works on boronic acid derivatives [14,17], and can be recovered through a more accurate treatment of relaxation effects for reconciling the theoretical results with the experiment.

Considering, in particular, the ΔSCF results, the main peak (A) is associated with the transition to the LUMO orbital, which has a predominant π(B–B) bonding character with minor π*(B–O) antibonding contributions, while peak C corresponds to the transitions to the antibonding π*(B–B) MO. This attribution agrees with the result found for the B_2_Cat_2_ diboron compound [14], as well as with the A–C energy separation, which is about 3.6 eV at ΔSCF level. The 3D plots of these π MOs are reported in Supplementary Figure S4. The low-intensity transitions in between (peak B) have a mainly diffuse character. The nature of the final states does not change in passing from ΔSCF to DFT-TP results; therefore, the assignment of the spectral features is confirmed by both approaches, as shown in Table 1. This is important in the perspective of the interpretation of the B1s DFT-TP spectra of the following extended models, for which the ΔSCF approach cannot be employed for their low symmetry, which prevents the SCF convergence of the core excited states.

### 3.2. M1, M2 and M3 Molecular Models

**Figure 3:**
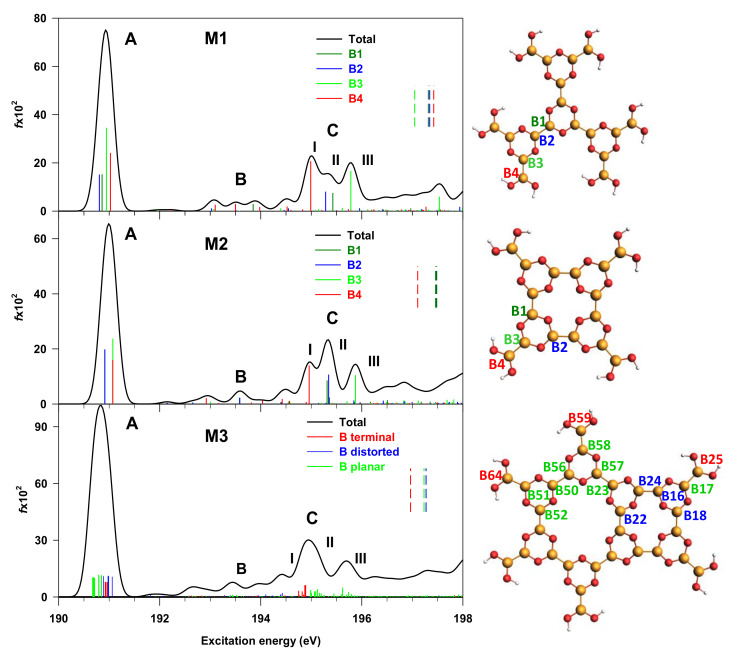
Calculated B1s NEXAFS spectra of M1 (**upper panel**), M2 (**middle panel**) and M3 (**lower panel**) molecular models depicted on the right side. Labels denote the non-equivalent B_i_ sites (see text for explanation). Transitions from the non-equivalent B_i_ atoms are reported as vertical colored lines in the spectra. The ΔSCF B1s ionization energies are shown as vertical dashed lines (the IP values are reported in Supplementary Table S1). The stick spectra are broadened by using a Gaussian line shape with FWHM = 0.3 eV.

The comparison between the B1s NEXAFS calculated spectra of M1, M2 and M3 boroxine-based molecular models is shown in Figure 3; the representation of the models with the labelling of the non-equivalent B centers (B_i_) is also reported.

It can be useful to underline that the boroxine rings are planar in the M1 model, while M2 is characterized by a certain strain and deviation from planarity; a significant strain is instead present in the M3 model, derived from the junction of two molecular fragments with different point group symmetry. Details on the identification of the non-equivalent B_i_ sites of each model are given in Section 3 in the Supplementary Materials. In particular, four B_i_ non-equivalent centers can be identified in the M1 and M2 models, whereas this number increases to fifteen in M3, as shown in Figure 3. In the M3 model, these non-equivalent B_i_ atoms can be grouped in three categories (as highlighted by the different colors in Figure 3): the terminal B_i_ atoms (in red), bonded to the –(OH)_2_ moieties, the planar B_i_ atoms (in green), which display a planar arrangement in the M3 model, the distorted B_i_ atoms (in blue), on which the largest strain in the M3 model, compared to M1 and M2, is localized.

Consider now a comparative analysis of the B1s NEXAFS spectra of the three models. For each system, a detailed attribution of the calculated transitions has been included in the Supplementary Materials (Tables S2–S4). The spectra of the three models are dominated by a first intense peak (A), whose energy position remains essentially constant (around 191 eV). Low-intensity features are present in the energy range between 192 and 194 eV (indicated with B), while a three-peaked structure (C) is present in the 194.5–196 eV energy range, which appears much more sensitive to the model structure compared to the other spectral features. Peak A has a common origin for the three models, being contributed by the B1s to LUMO transitions, starting from all the non-equivalent B_i_ atomic centers towards the LUMO orbital (see Supplementary Table S2). The increasing number of B_i_ non-equivalent atoms in going from M1 to M3 leads to the splitting of the four transitions of M1 and M2 into several closely spaced transitions in M3, whose relative spacing reflects the different geometrical environment of the B_i_ non-equivalent centers. Further, the nature of the LUMO orbital is conserved along the series: it is characterized by a π(B_i_–B) bonding and π*(B_i_–O) antibonding character between the B_i_ excited atom and the O atom bonded to it (see panel (a) of Supplementary Figures S5–S7). Both the energy position (around 191 eV) and nature of peak A still agree with the results found for the diboron compounds THDB, above described, and for the B_2_Cat_2_ molecule of ref. [14]. The transitions of B features in all the three models have a very low intensity as they involve final MOs of mainly π(B–B) bonding character relative to B atoms close to the B_i_ excited site.

Feature C is the most sensitive to molecular structure modification; in fact, it evolves from a three-peaked structure in M1 and M2 to a broader one in M3. This evolution is partially due to the increased number of the B_i_ non-equivalent centers found in going from M1 to M3; however, the increased molecular strain of model M3 compared to M1 and M2 also has a role, as explained in the following. Feature C of the M1 and M2 spectra derives from transitions from all the four non-equivalent B_i_ atoms towards orbitals of mainly π*(B_i_–B) antibonding character. In particular, sub-peaks I and III are assigned to the π*(B_i_–B) antibonding external bonds (involving the B4 and B3 sites) (see Supplementary Figures S5 and S6), while sub-peak II is attributed to the π* antibonding transitions from the B_i_ atoms involved in the B–B bonds between two boroxine rings. The transitions from the B_i_ external site (B4) give the major contribution to the lower-energy side of peak C (at about 195 eV) in both models; therefore, the slight distortion from planarity of M2 compared to M1 does not significantly influence the nature and energy of the B1s transitions.

More significant differences affect, instead, feature C of the M3 spectrum, compared to M1 and M2; in particular, the sub-peaks are broader and less resolved for the presence of a large number of transitions from the many non-equivalent B_i_ atoms, over which the intensity redistributes. The structural nature of the B_i_ atoms of M3 is useful to analyze feature C, keeping in mind that the planar B_i_ atoms (in green) are involved in B–B bonds with the terminal B_i_ atoms (external bonds), as well as in B–B bonds between boroxine rings (see lower panel of Figure 3). Sub-peak C(II) is the most intense one, and is contributed by transitions from the terminal (red lines) and planar (green lines) B_i_ sites involved in the B–B external bonds, while sub-peak C(III) transitions from the remaining planar B_i_ atoms forming the B–B bonds between boroxine rings. Therefore, these transitions gather together in two sub-peaks, according to the B–B bond’s nature, in analogy with what was found for the transitions of peak C in the M1 and M2 spectra. The final MOs of C(II) and C(III) transitions still have a mainly π*(B_i_–B) antibonding character (see Supplementary Table S4). Of particular interest is sub-peak C(I), which is associated with transitions from the B_i_ distorted atoms; the latter are involved in B–B bonds among boroxine rings belonging to the square moiety of M3, where the larger molecular strain is present. The final MOs are mostly localized over the square moiety (see panel (c) in Figure S7), and have a mainly π(B–B) character, not involving the excited B_i_ atoms, with a consequent suppression of the intensity, compared to the other two groups of B_i_ atoms in M3.

In summary, the most significant insights emerging from the evolution of the B1s NEXAFS features from the THDB precursor to the M1, M2 and M3 models concern: (a) the consistency in terms of energy position and ratio intensity of the A and C features, assigned to the π(B–B) bonding and antibonding orbitals, respectively, which, therefore, represent a probe in the presence of the B–B bond in the systems; (b) the evolution of the C feature along the series as the number of the non-equivalent B_i_ centers increases and their geometrical environment changes due to molecular strain and distortion from planarity.

### 3.3. M3@Au(111) Model

**Figure 4:**
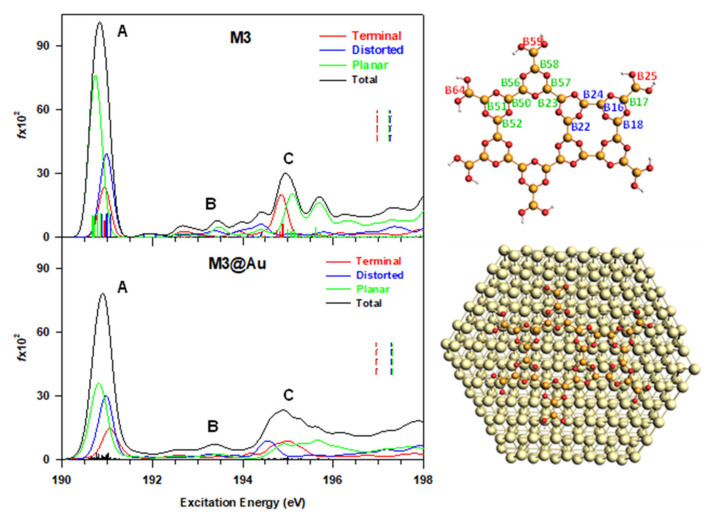
Comparison between the calculated B1s NEXAFS spectra of free M3 (**upper panel**) and M3@Au(111) (**lower panel**). The contributions of the three groups of non-equivalent B_i_ sites are also shown (colored solid lines). The ΔSCF B1s ionization energies are shown as vertical dashed lines (the IP values are reported in Supplementary Table S1). The stick spectra are broadened by using a Gaussian line shape with FWHM = 0.3 eV. The M3 and M3@Au(111) models employed are reported on the right side.

The results of the calculated B1s NEXAFS spectrum of M3 adsorbed on the Au(111) surface are reported in Figure 4 and compared with the results of the isolated M3 model. The top view of the cluster cut out from the periodic relaxed structure is also shown; note that the numbering and classification of the non-equivalent B_i_ sites are the same employed for the isolated M3 model.

The similarity of the two profiles is well evident and the energy position of the peaks is preserved, confirming a weak electronic coupling between the substrate and the boron atoms in the adsorbed M3 system. The reduction in the first peak intensity upon the adsorption can be ascribed to the redistribution of the intensity among a larger number of transitions, compared to free M3, due to a certain degree of orbital rehybridization with the Au substrate *sp* states. The final MOs involved are largely localized on the adsorbate and maintain a mostly π nature. The three sub-peaks of the C band around 195 eV of free M3 model collapse on a single, smooth feature in M3@Au, whose transitions still distribute according to the structural nature of the B_i_ atoms: transitions from the distorted B_i_ atoms fall at lower energy, the intermediate region is assigned to planar and terminal B_i_ atoms, while the portion at higher energy is associated with the remaining planar B_i_ atoms.

**Figure 5:**
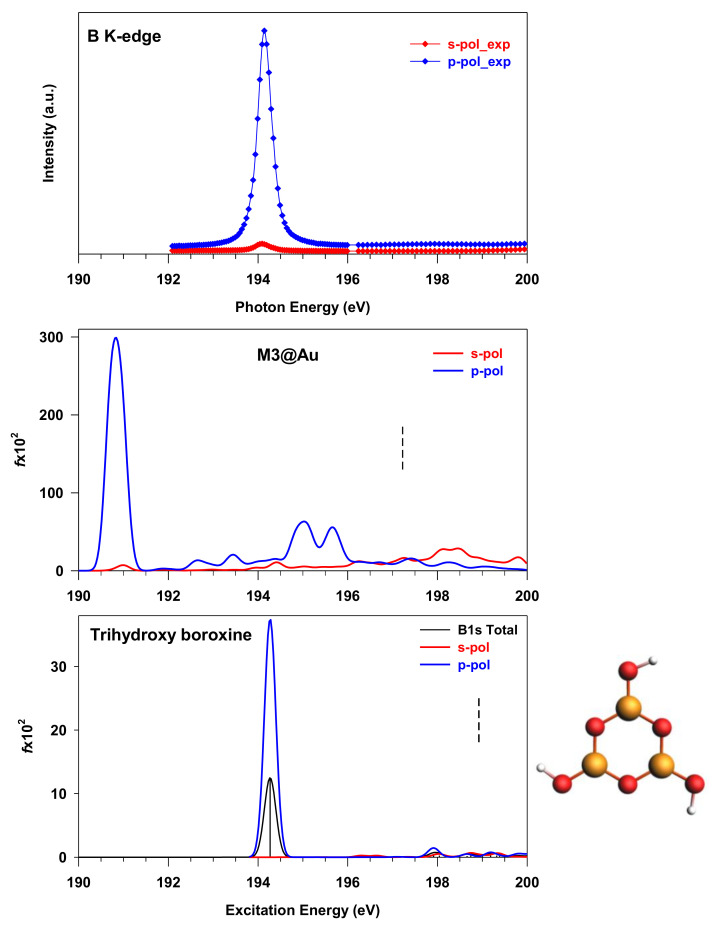
B1s NEXAFS spectra of boroxinated monolayer ((**upper panel**), experimental data with permission from ref. [7]), M3@Au(111) (**middle panel**) and THBoroxine (**lower panel**) at the two different polarization angles. For the THBoroxine molecule (displayed along the corresponding spectrum), the B1s total spectrum is also reported. The ΔSCF B1s ionization energies are shown as vertical dashed lines (IP mean value for M3@Au, 198.92 eV for THBoroxine). The calculated stick spectra are broadened by using a Gaussian line shape with FWHM = 0.3 eV.

Aiming at comparing the theoretical results with the experiment of ref. [7], the s- and p-pol spectra of M3@Au have also been calculated, considering the two light polarizations used for the acquisition of the experimental spectra; mainly, parallel to the surface (s-pol) and normal to the surface (p-pol). In the reference system used for the calculations, the Au surface lies in the xy plane and z is the normal direction. The B1s experimental spectra present a large dichroism between the two polarizations, traced to a closely planar orientation in the network on the Au surface. A single sharp peak is observed around 194 eV in the p-pol spectrum, while at higher energies, both profiles are flat (see Figure 5).

The theoretical s- and p-pol spectra capture the dichroism of the first peak; however, two main discrepancies with the experiment are evident—mainly, the calculated energy position of the first peak (190.8 eV) and the presence of a feature around 195 eV (indicated as C in Figure 4), which is not visible in the experimental p-pol profile. The energy of the calculated main peak is about 4 eV lower than in the experiment, and such a difference cannot be ascribed to a deficiency of calculations, but rather to non-effective modeling of the monolayer structure condensed on the surface. The molecular models are, in fact, based on the hypothesis of a network of B–B-bonded boroxine groups in different geometries of connectivity, and all the calculated B1s NEXAFS spectra show two structures of π(B–B) bonding and antibonding nature, consistent with the presence of B–B bonds, as found for the B_2_Cat_2_ [14] and THDB diboron molecules. To further explore the possible origin of the discrepancies between theory and experiment, we tentatively considered a boroxine-based molecule, in which the boron is in a different chemical environment. To this aim, we selected the THBoroxine molecule, whose structure is shown in the lower panel of Figure 5, together with the corresponding B K-edge spectrum calculated at DFT-TP level. In the THBoroxine molecule, boron atoms in the ring are not involved in a bonding with another B atom, but with a more electronegative O atom in the –OH group. Only one main peak is obtained at 194.27 eV, corresponding to the transition to the LUMO orbital, which is an anti-bonding π*(B–O) orbital contributed by the 2p perpendicular atomic components of B and O atoms in the ring, and, to a lesser extent, in the –OH terminations (see Supplementary Figure S8). The s- and p-pol contributions, calculated by considering the molecule fixed in space, are also shown, and confirm the π nature of the peak.

Considering the energy position of the π(B–B) bond transitions of the previous systems (around 191 eV), a significant increase in the excitation energy is obtained when the B atoms of the boroxine are involved in external B–OH bonds in place of B–B bonds. The obtained spectrum resembles the one measured and calculated for the B(OH)_3_ molecule in its gas phase [17]. The observed trend in the excitation energy can be related to the decrease in electronic density on the B atoms of the boroxine ring bonded to the –OH fragments in the THBoroxine compared to the M1-bonding situation, and supported by our Bader’s population analysis. The depletion of electron density away from the boroxine B atoms is responsible for the increased B1s binding energy of THBoroxine, compared to the other B–B bond systems, as well as of the excitation energy of the B1s to LUMO transition. We finally point out that a slightly lower energy is calculated at ΔSCF level for the B1s to LUMO transition (193.52 eV) of THBoroxine, with a trend already found for the THDB molecule.

Figure 5 highlights that the THBoroxine spectrum is similar both in shape and in peak energy position with the experimental one of the boroxine framework [7]. This suggests that the boron atoms in the two systems are in a similar chemical configuration. One possible explanation is that the boroxine groups in the experimental framework are bonded through B–O bonds instead of B–B bonds. This could happen due to the Au-catalysed cleavage of the B–B bond of the precursor molecule, before or after the condensation of boroxine rings, followed by a whole re-arrangement of the rings to form B–O bonds between each other. Even if we cannot exclude this growth mechanism in the framework, its complexity from the kinetic point of view suggests that we should explore other possibilities to explain the spectroscopic similarities with the THBoroxine. An alternative explanation is that the interaction with gold promotes a charge transfer from the B–B bond to the surface, so that the depletion of charge around the B atoms in the framework could resemble the electronic configuration described in the case of the B–OH bond present in the THBoroxine. Evidently, this charge transfer effect, which strongly affects the nature of the B–B bond, is not present in our M3@Au model. Further investigation is needed to reveal the possible activation of a stronger interaction of the B–B species with the substrate through the building of suitable molecular models to describe it.

## 4. Conclusions

The present computational study aimed to identify possible building block molecules, able to model the boroxine 2D framework obtained in ref [7] from the condensation of the THDB molecule after deposition on the Au(111) surface. Three different models of increasing complexity (M1, M2 and M3) have been proposed, based on different boroxine rings arrangement through B–B bonds. The molecular models have been electronically characterized by means of DFT-TP calculations in the B1s NEXAFS spectra, focusing on the role on the spectral features of the different geometries of the B–B bonds and the progressive inclusion of defects in the model. The THDB precursor has been considered as well as used to test the influence on the B1s spectrum of the more accurate treatment of relaxation effects, compared to the DFT-TP scheme, through ΔSCF calculations, which cannot be performed for the larger model systems. The calculated B1s spectra do not significantly change along the series and are characterized by two main features (indicated as A and C), deriving from the transitions to π(B–B) bonding and antibonding final orbitals, respectively, and can be considered a fingerprint of the presence of the B–B bond, according to previously published results on the diboron B_2_Cat_2_ system [14]. The simulation of B1s spectrum of the more complex model M3 adsorbed on the Au (111) surface (M3@Au) does not show significant differences with that of the free M3; therefore, a weak electronic coupling between the substrate and the B atoms of the adsorbed M3 system emerges from the calculations. The comparison of the M3@Au calculated spectrum with the B K-edge experimental spectrum reveals important discrepancies and points out that the considered models are not able to capture the nature of the boroxine framework on the Au surface. Possible suggestions for further studies come from the results obtained for the B1s spectrum of the THBoroxine molecule, which show that the change in the chemical environment of boroxine B atoms bonded to terminal –(OH) groups give rise to a single spectral feature, in agreement with that experimentally observed. This aspect points to either a breaking of the B–B bonds in the framework, with a consequent re-arrangement of the boroxine groups, or to a stronger interaction between the boroxine framework and the substrate, possibly leading to the weakening of the B–B bond, whose nature requires further investigation.

## Figures and Tables

**Table 1:** Calculated B1s NEXAFS spectrum of THDB: ΔSCF and DFT-TP transition energies (in eV) and oscillator strengths (f × 10^2^).

Peak	Transition	ΔSCF Results		DFT-TP Results		Assignment, Main Character of the Final MO
		E(eV)	f × 10^2 a^	E(eV)	f × 10^2 a^	
A	B1s → 5a”	191.13	6.74	191.65	10.56	π(B–B) + π*(B–O)
B	B1s → 15a’	193.22	0.50	194.16	0.66	mixed valence σ*(B–B), σ*(O–H)/Rydberg
	B1s → 16a’	193.71	0.61	194.72	0.74	mixed valence σ*(O–H)/Rydberg
C	B1s → 6a”	194.69	1.37	195.73	5.62	π*(B–B) + π*(B–O)
	B1s → 7a”	195.22	1.54	196.10	2.10	π*(B–B) + π*(B–O)

^a^ Only calculated transitions with f × 10^2^ ≥ 0.50 are reported.

## Data Availability

Data are contained within the article.

## Supplementary Materials

The following supporting information can be downloaded at: https://www.mdpi.com/article/10.3390/nano12091610/s1, Figure S1: Ideal 2D boronic network from THDB precursor; Figures S2 and S3: Representation of the unit cells employed for the geometry optimization of the systems; Figures S4–S8: 3D plots of selected molecular orbitals of the systems; Table S1: Calculated IP potentials of the model systems; Tables S2–S4: Peak assignments of the total B1s NEXAFS spectra of M1, M2 and M3 molecular models with a short description of their geometrical structure.
