# Supplementary material for: Computational NEXAFS Characterization of Molecular Model Systems for 2D Boroxine Frameworks
Source: Nanomaterials (Basel). 2022 May 9;12(9):1610. doi: 10.3390/nano12091610 (PMC9100003; doi:10.3390/nano12091610)
Supplement: Supplementary file 1 [file nanomaterials-12-01610-s001.zip › nanomaterials-1673649-supplementary.pdf]

nanomaterials

Supplementary Material

Computational NEXAFS Characterization of Molecular Model Systems for 2D Boroxine Frameworks

Daniele Toffoli [1], Elisa Bernes [1], Albano Cossaro [1,2], Gabriele Balducci [1], Mauro Stener [1], Silvia Mauri [1] and Giovanna Fronzoni [1,*]

[1] Department of Chemical and Pharmaceutical Sciences, University of Trieste, Via L. Giorgieri 1, I-34127 Trieste, Italy; toffoli@units.it (D.T.); elisa.bernes@units.it (E.B.); acossaro@units.it (A.C.); balducci@units.it (G.B.); stener@units.it (M.S.); silvia.mauri@phd.units.it (S.M.)

[2] CNR-IOM, Istituto Officina dei Materiali, I-34149 Trieste, Italy

* Correspondence: fronzoni@units.it

1. Systems and geometry optimization

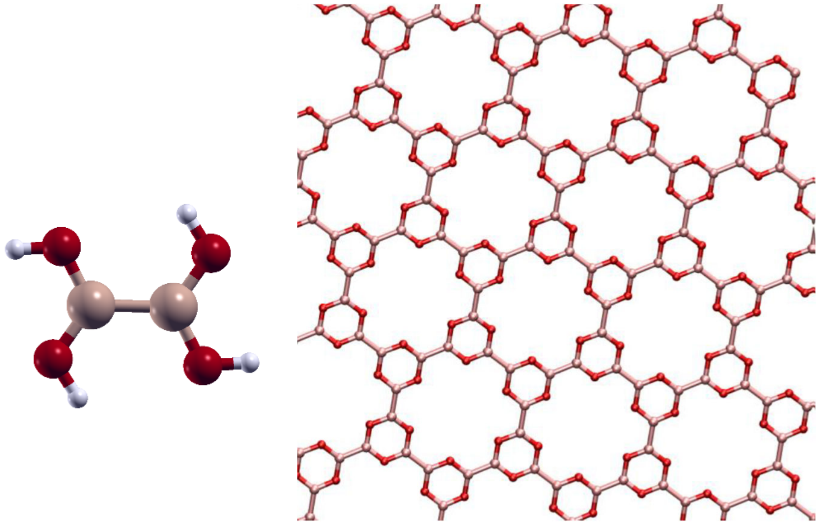

Figure S1. Left: THDB molecule. **Right**: representation of the ideal 2D boronic network from the THDB precursor, the 6-fold boroxine assemblies are well recognizable.

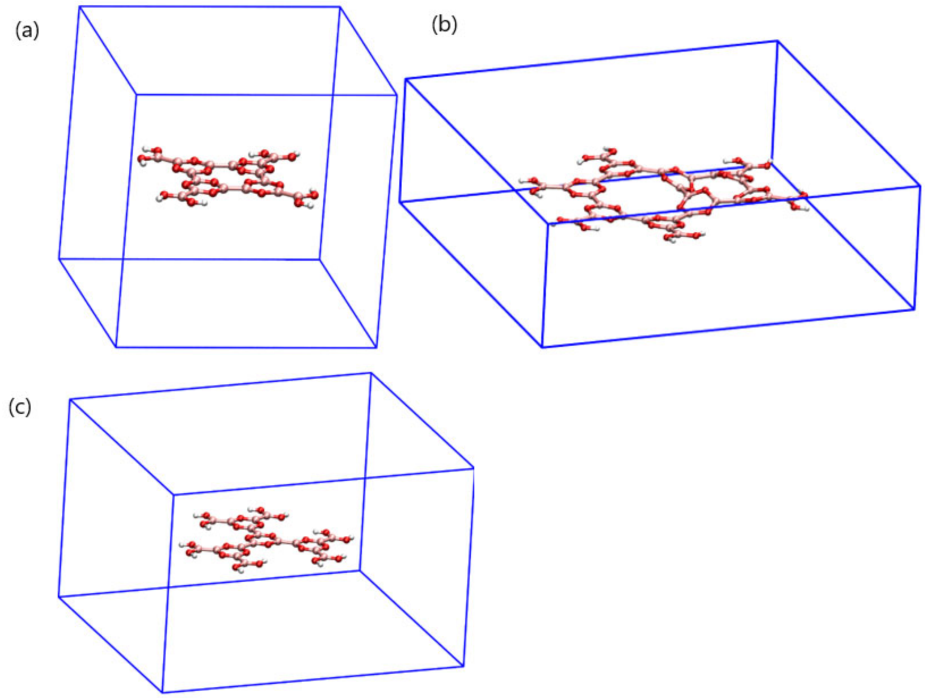

Figure S2. Representation of the unit cells employed in the geometry optimization of the isolated molecular models: (**a**) M1; (**b**) M2; (**c**) M3.

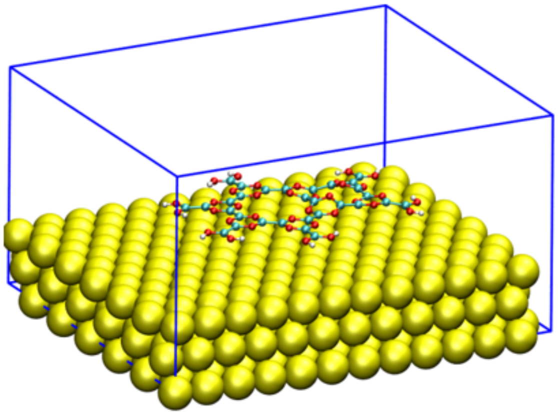

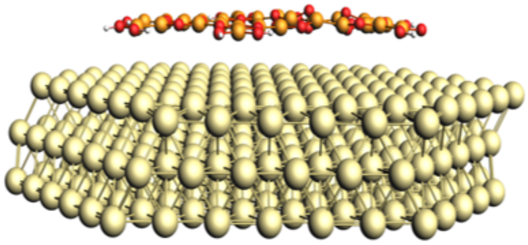

Figure S3. Left: representation of the unit cell employed in the geometry optimization of the M3@Au model. **Right**: side view of the cluster model cut out from the relaxed periodic structure.

2. 3D plots of final MOs corresponding to selected ΔSCF B1s core excited transitions of THDB

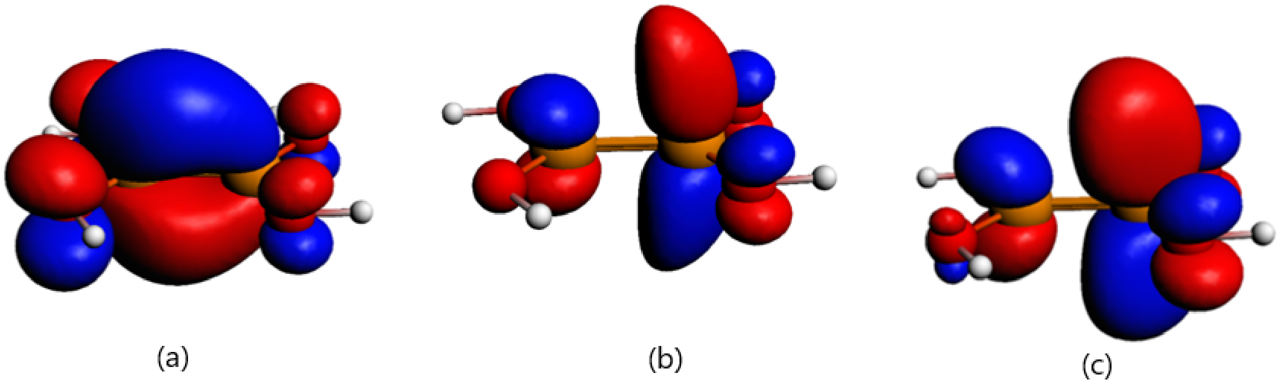

Figure S4. 3D plots of selected final MOs of THDB relative to the ΔSCF B1s core excitation calculations: (**a**) 5a″ (transition at 191.13 eV); (**b**) 6a′′ (transition at 194.69 eV); (**c**) 7a″ (transition at 195.22 eV). Displayed isosurface corresponds to ± 0.035 $e^{1/2}a_0^{-3/2}$ value**.**

3. B1s NEXAFS theoretical results

Table S1. ΔSCF IP potentials (in eV) for the M1, M2, M3 and M3@Au models.

Model	B1	B2	B3	B4
M1	197.42	197.34	197.32	197.05
M2	197.46	197.47	197.48	197.11
	B planar*	B distorted*	B terminal*	
M3	197.23	197.27	196.96	
M3@Au	197.30	197.27	196.96	

*IPs mean value

3.1. M1 model

The M1 model (see figure below from Figure 3 of the manuscript)

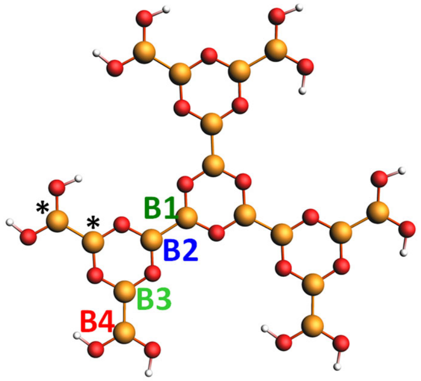

has been assumed to belong to a pseudo-C_{3h} point group, since its structure is not perfectly planar; indeed, in an ideal planar structure, a C_3 rotation axis passing through the center of the inner ring of the molecule would be present. In this perspective we identified the 4 non-equivalent B_i centers highlighted in the Figure. Since there is a certain degree of bending, this assumption was checked by performing preliminary calculations of NEXAFS spectra of the remaining two B centers, indicated with an asterisk in the Figure.

Table S2 can be used as a guide to follow the assignment of the B1s NEXAFS spectrum of M1 (reported in the upper panel of Figure 3 of the manuscript).

Table S2. Peak assignments of the total B1s NEXAFS spectrum of the M1 model. DFT-TP excitations energies (in eV) and oscillator strengths ($f \times 10^2$) are reported.

Peaks	B_i core-hole site	E(eV)	$^a f \times 10^2$	Assignment, main character of the final MO
A	**B2**	**190.80**	**15.2**	LUMO $\pi(B_i\text{–}B) + \pi^*(B_i\text{–}O)$
	B1	190.86	15.3	
	B3	190.94	34.6	
	B4	191.02	24.1	
B	B2	193.10	1.18	$\pi(B\text{–}B) + \pi^*(B_i\text{–}B)$ (minor)
	B4	193.11	2.68	
	B4	193.50	3.01	
	B1	193.85	3.01	
	B4	194.52	1.92	
	B2	194.54	1.18	
C(I)	B4	194.99	20.7	$\pi^*(B_i\text{–}B) + \pi^*(B_i\text{–}O)$
C(II)	B2	195.28	8.09	
	B1	195.43	7.53	
C(III)	B3	195.61	1.36	Rydberg
	B3	195.68	16.7	$\pi^*(B_i\text{–}B) + \pi^*(B_i\text{–}O)$

[a]Only transitions with $f \times 10^2 > 1.15$ are reported.

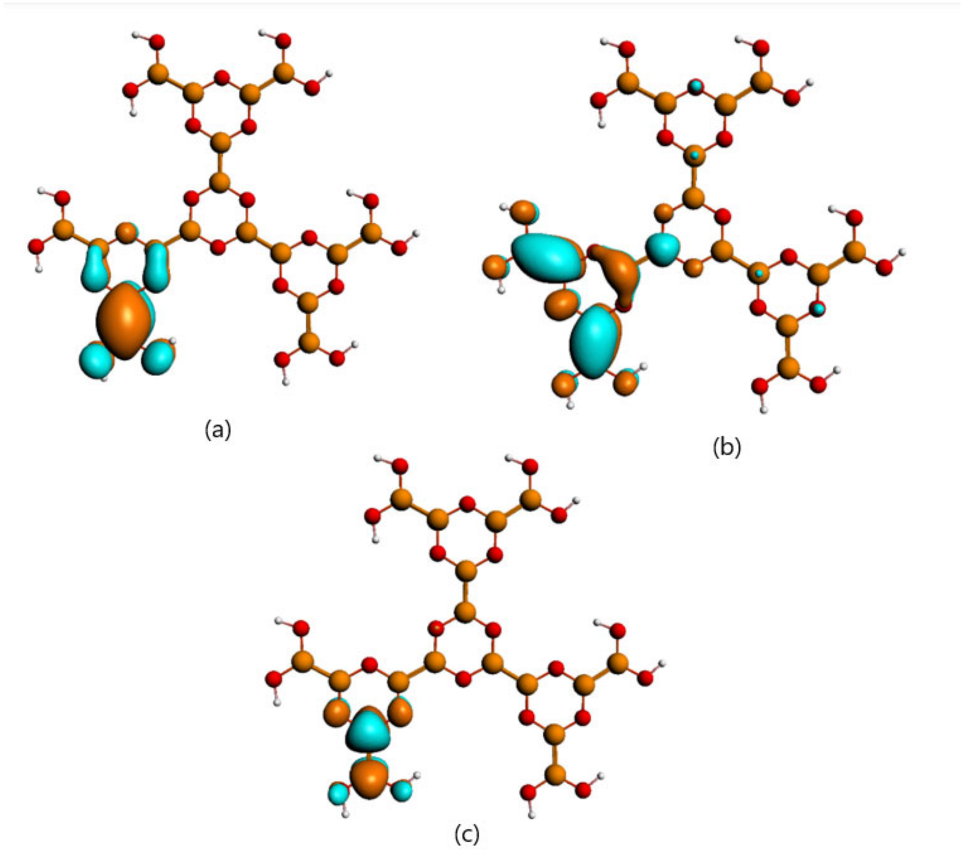

Figure S5. 3D plots of selected final MOs of model M1 relative to the DFT-TP B1s core excitation calculations: (**a**) LUMO, B4 core-hole (transition at 191.02 eV); (**b**) LUMO+6, B1 core-hole (transition at 193.85 eV); (**c**) LUMO+12, B4 core-hole (transition at 194.99 eV). Displayed isosurface corresponds to ±0.030 for (**a**) and (**b**), and 0.038 for (**c**) $e^{1/2}a_0^{-3/2}$ value**.**

3.2. M2 model

The M2 model (see figure below from Figure 3 of the manuscript)

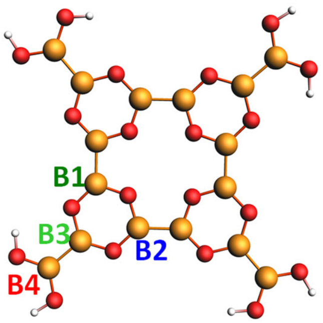

has been assumed to belong to a pseudo-C_{4h} point group, since its structure is not perfectly planar; indeed, in an ideal planar structure, a C_4 rotation axis and an inversion center *i* passing through the center of the inner ring of the molecule would be present. Based on this hypothesis, the four non-equivalent B_i core-hole sites labelled in the figure have been identified.

Table S3 can be used to analyze the B1s NEXAFS spectral features of M2 (see middle panel of Figure 3 of the manuscript).

Table S3. Peak assignments of the total B1s NEXAFS spectrum of the M2 model. DFT-TP excitations energies (in eV) and oscillator strengths (f×10^2) are reported.

**Peaks**	**B_i core-hole site**	**E(eV)**	**^{a}f×10^2**	**Assignment, main character of the final MO**
A	**B2**	**190.91**	**19.8**	π(B_i–B) + π*(B_i/B–O)
	B1	190.91	19.6	
	B3	191.07	23.7	
	B4	191.07	16.0	
B	B4	192.92	2.11	π(B–B) + π*(B_i–B) (minor)
	B1	193.58	2.28	
	B2	193.58	2.31	
	B4	194.42	1.89	
C(I)	B4	194.96	14.0	π*(B_i–B) + π*(B_i/B–O)
C(II)	B1	195.31	8.58	
	B2	195.34	10.7	π*(B_i–B) + π*(B_i/B–O)/Rydberg
	B1	195.36	2.40	
C(III)	B3	195.87	10.5	π*(B_i–B) + π*(B_i/B–O)

^{a}Only transitions with f × 10^2>2.00 are reported.

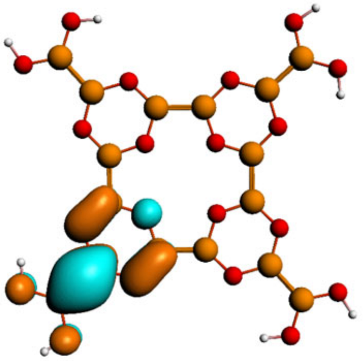
(a)

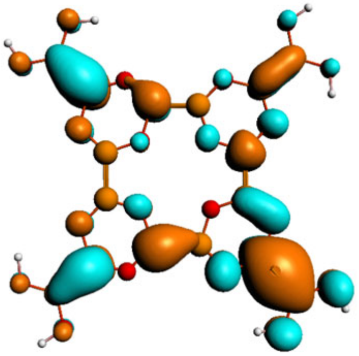
(b)

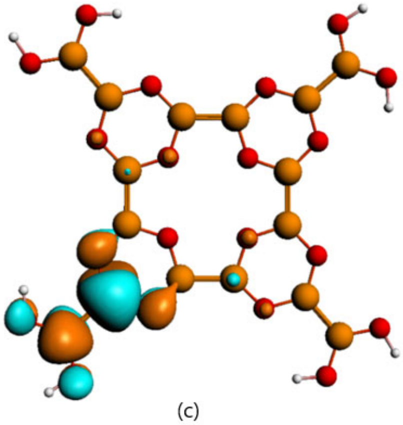
(c)

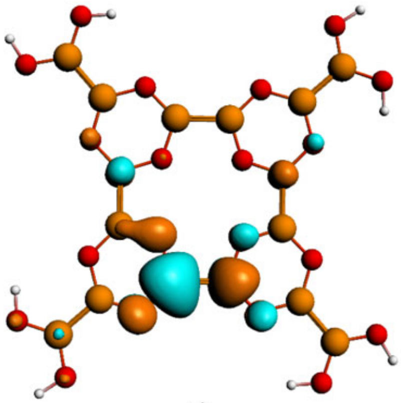
(d)

Figure S6. 3D plots of selected final MOs of model M2 relative to the DFT-TP B1s core excitation calculations: (**a**) LUMO, B3 core-hole (transition at 191.07 eV); (**b**) LUMO+5, B2 core-hole (transition at 193.58 eV); (**c**) LUMO+11, B4 core-hole (transition at 194.96 eV); (**d**) LUMO+11, B2 core-hole (transition at 195.34 eV). Displayed isosurface corresponds to ±0.030 for (**a**) and (**c**), 0.020 for (**b**) and 0.032 for (**d**) $e^{1/2}a_0^{-3/2}$ value**.**

3.3. M3 model

The M3 model (see figure below from Figure 3 of the manuscript)

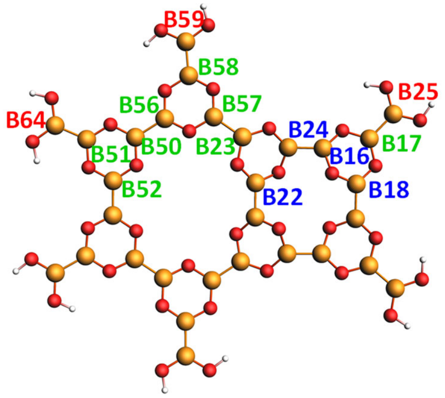

has been assumed to have a *pseudo*-C_2 symmetry, since its structure slightly diverges from planarity; if we assume an ideal planar structure, a C_2 rotation axis passing through the center of the square and hexagon would exist. Based on this assumption we identified fifteen non-equivalent B_i sites which are indicated in the figure above and subdivided into three groups, including terminal (in red), distorted (in blue) and planar (in green) atoms, according to the corresponding position and arrangement in the molecule.

The B1s NEXAFS transitions of M3 can be analyzed by using Table S4 (see lower panel of Figure 3 of the manuscript for the corresponding NEXAFS spectrum).

Table S4. Peak assignments of the total B1s NEXAFS spectrum of the M3 model. DFT-TP excitations energies (in eV) and oscillator strengths (f × 10^2) are reported.

**Peaks**	**B_i core-hole site**	**E(eV)**	**^{a}f×10^2**	**Assignment, main character of the final MO**
	**B52**	**190.67**	**10.2**	
	B23	190.68	10.5	
	B56	190.69	10.1	
	B50	190.71	10.2	
	B57	190.71	10.0	
	B51	190.79	11.4	
	B17	190.80	11.8	
A	B58	190.85	11.5	LUMO/π(B_i–B) + π*(B_i–O)
	B18	190.89	10.9	
	B25	190.92	7.91	
	B64	190.93	7.93	
	B59	190.96	7.90	
	B24	190.98	11.0	
	B16	190.99	11.2	
	B22	191.06	11.7	

B	B59	192.58	0.67	π(B–B) + π*(B–O)
	B25	192.59	0.41	
	B64	192.63	0.68	
	B24	193.28	0.95	
	B18	193.39	0.96	
	B23	193.52	0.98	
C(I)	B22	193.95	1.93	π(B-B)
	B16	194.10	1.37	
	B16	194.23	0.79	
	B24	194.40	1.51	
	B18	194.43	2.18	
C (II)	B25	194.75	3.31	π*(B$_i$–B) + π(B–B)
	B25	194.82	2.98	
	B59	194.85	1.24	Rydberg
	B64	194.87	6.26	π*(B$_i$–B) + π*(B–O)
	B59	194.89	6.25	
	B57	194.99	3.78	
	B23	195.00	2.24	mixed valence π*(B$_i$–B) + π*(B–O)/Rydberg
	B52	195.06	2.87	
	B56	195.09	3.12	
	B50	195.13	4.13	
	B52	195.17	2.19	
	B56	195.20	1.85	
C (III)	B23	195.45	1.81	mixed valence π*(B$_i$–B) + π*(B–O)/Rydberg
	B58	195.60	1.47	
	B51	195.62	4.94	
	B17	195.69	1.22	
	B58	195.73	2.64	
	B17	195.80	1.51	

[a] Only transitions with f × 10^2>0.40 are reported.

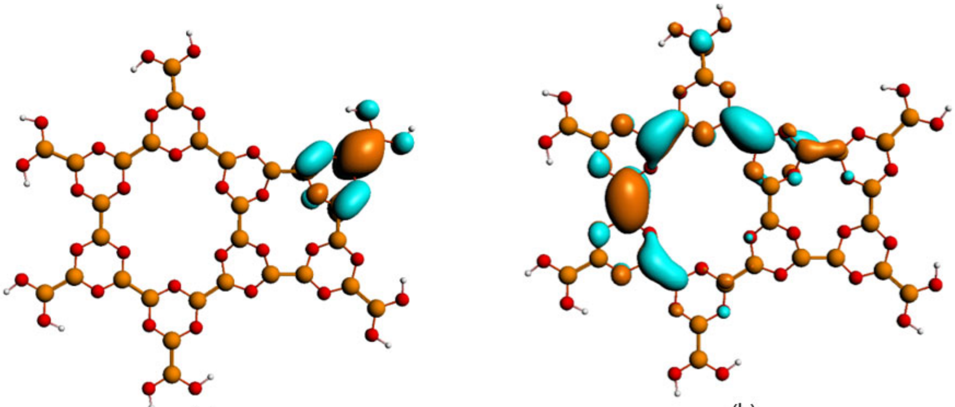

(a) (b)

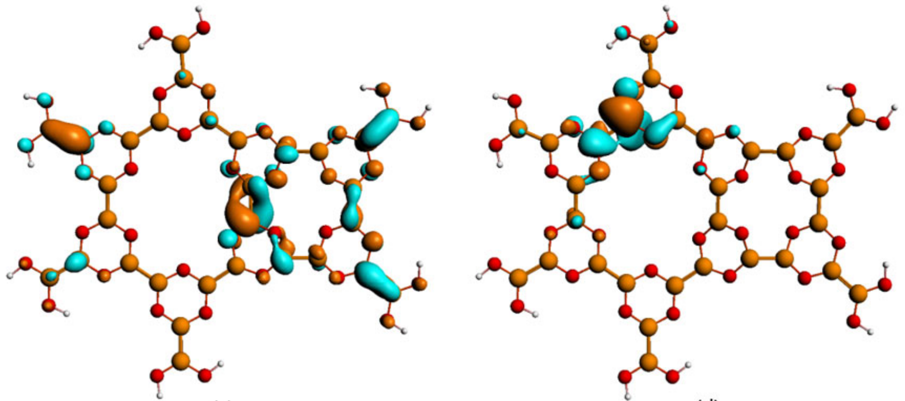

Figure S7. 3D plots of selected final MOs of model M3 relative to the DFT-TP B1s core excitation calculations: (**a**) LUMO, B17 core-hole (transition at 190.80 eV); (**b**) LUMO+2, B59 core-hole (transition at 192.58 eV); (**c**) LUMO+12, B22 core-hole (transition at 193.95 eV); (**d**) LUMO+15, B50 core-hole (transition at 195.13 eV). Displayed isosurface corresponds to ±0.030 for (**a**), (**c**) and (**d**) and 0.025 for (**b**) $e^{1/2}a_0^{-3/2}$ value **.**

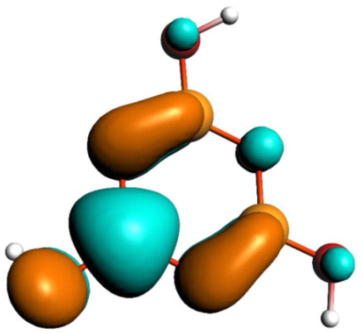

Figure S8. 3D plot of the LUMO orbital of the THBoroxine relative to the DFT-TP B1s core excitation calculations. Displayed isosurface corresponds to ±0.030 $e^{1/2}a_0^{-3/2}$ value.
